# Vertical integration in medical education: the broader perspective

**DOI:** 10.1186/s12909-020-02433-6

**Published:** 2020-12-14

**Authors:** Marjo Wijnen-Meijer, Sjoukje van den Broek, Franciska Koens, Olle ten Cate

**Affiliations:** 1 Technical University of Munich, TUM School of Medicine, TUM Medical Education Center, Ismaninger Straße 22, 81675 Munich, Germany; 2 University Medical Center Utrecht, School of Medicine, Utrecht, The Netherlands; 3 Amsterdam UMC, Faculty of Medicine Vrije Universiteit Amsterdam, Amsterdam, The Netherlands; 4 University Medical Center Utrecht, Center for Research and Development of Education, Utrecht, The Netherlands

## Abstract

Curricular integration represents collaborations between disciplines to establish a coherent curriculum and has become the dominant recommendation for medical education in the second half of the twentieth century. Vertical integration specifically is the integration between the clinical and basic science parts throughout the program. Vertically integrated curricula present basic sciences imbedded in a clinical context from the start of medical school.

The authors briefly discuss vertical integration in relationship with context theory, motivation theory, professional identity formation, transition to practice and the continuum of education and practice. They conclude that vertical integration, rather than horizontal integration, extends far beyond curriculum structure. They consider vertical integration a philosophy of education, with impact on students’ maturation and engagement with the profession, and which applies not only to undergraduate education but to the lifelong learning of professionals. The definition of vertical integration as “an educational approach that fosters a gradual increase of learner participation in the professional community through a stepwise increase of knowledge-based engagement in practice with graduated responsibilities in patient care” is more comprehensive than its older conceptualization.

## Integration in medical education

Integration has been a popular concept in medical education for decades. An integrated curriculum, focusing on general objectives of medical education, rather than a curriculum merely composed of a stack of separate courses and clerkships with their own culture, professors, rules, and exams, featured in Harden’s SPICES model in 1984 as a key component (the *I*) of modern medical education [1]. But curricular integration has been advocated long before that important publication. In the 1950s, the Association of American Medical Colleges (AAMC) specifically stated that the objectives of undergraduate medical education should *not* include detailed systematic knowledge of the various basic sciences separately anymore. Instead, the AAMC spelled out a list of general goals for the medical school curriculum, all focused on learning necessary knowledge and basic skills related directly to patient care and not as ends in themselves [2]. In the decades to follow, many medical schools made curricular revisions offering forms of integration.

An excellent description of today’s use of integration in medical education is provided by Brauer & Fergusson [3]. The authors describe *horizontal integration* as the “integration across disciplines but within a finite period of time” [3], usually referring to the basic sciences. *Vertical integration* (VI) means integration across time. In a vertically integrated curriculum the time spent on classroom education gradually decreases across the years, while the amount of clinical practice increases [3], implying a move away from a strict caesura between theory and practice. Early experience with clinical problems and in clinical settings is interspersed with continued science teaching, be it less and less over time. In contrast, in a traditional curriculum classroom teaching is programmed in the first years of medical school, and clinical training in the final years. Vertical integration, without using the word, was advocated in 1980 as “gradually changing from [a curriculum] with a major emphasis on the basic medical sciences to one with a major emphasis on clinical medicine” [4]. These innovations had profound consequences for the curricular structure, as they integrated worlds that were traditionally, and still are in many programs worldwide, disconnected. Problem-based learning, as an example, is a pedagogical approach that is often used with the aim of creating curricular integration [5].

The aim of vertical integration is to support meaningful learning. VI curricula provide relevance to basic sciences for clinical practice, by matching learning with the way the knowledge is to be used [3]. This supports an important aspect of adult learning theory explaining adult learners’ focus on the relevance of the topic when learning [6]. In addition, it has been suggested that similarity between the context in which something is learned and in which that knowledge is to be applied enhances memory retrieval [7], and that the connection between basis science and clinical cases stimulates the building of relevant knowledge frameworks [8, 9]. Indeed, contemporary instructional design theory advocates training students in integrated, whole tasks rather than in separate, seemingly independent components that learners are expected to integrate by themselves [10, 11]. There is some criticism on VI, in addition to practical difficulties inherent to a major curriculum reform when creating a VI curriculum [12]. A discipline, taught scattered at many fragmented moments during the curriculum to serve the integration with other disciplines, may undermine its big picture and internal logic for students [13]. An example is pharmacology education, traditionally one of the more challenging classes. Adding scattered bits of pharmacology to various clinical education courses creates better embedding, but also risks, if students can neglect pharmacology without failing integrated tests, when the proportion of pharmacology in each test is small. Such issues are curricular construction problems that need to be addressed with practical solutions.

## A broader perspective on vertical integration

**Fig. 1:**
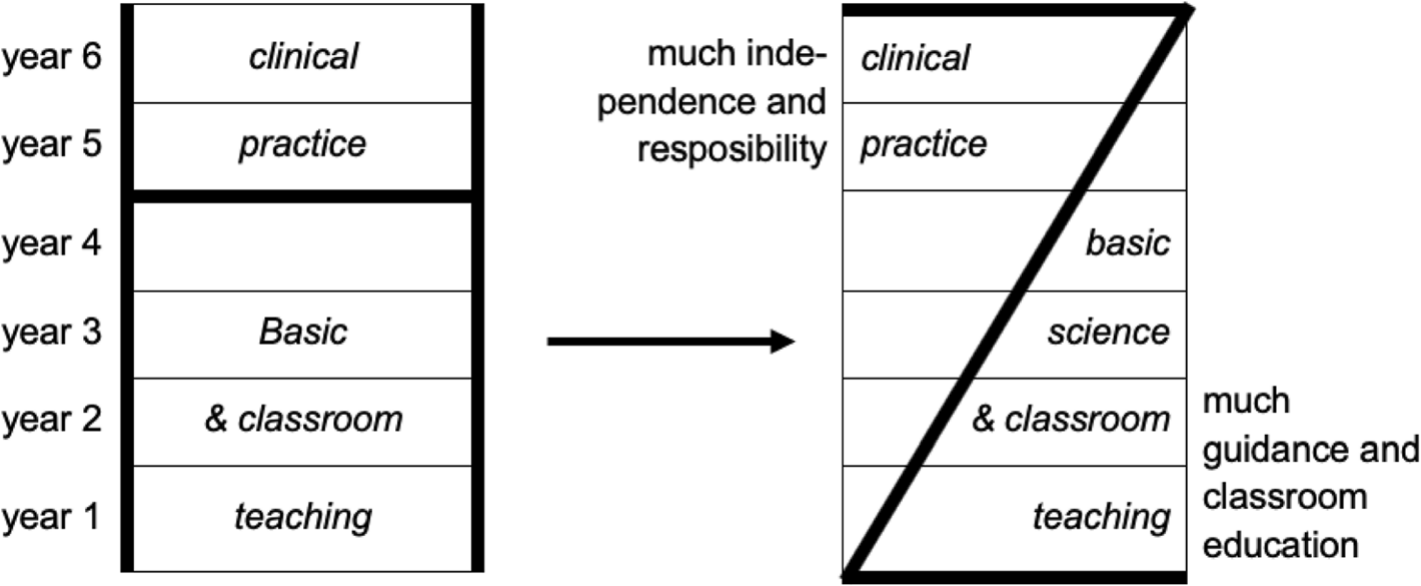
Vertical integration: The traditional H-shaped medical curriculum is being replaced by a Z-shaped curriculum model (from [14] with permission)

Vertical integration in medical education may be viewed as a reorganized curriculum structure (as a move from an H to a Z shape - see Fig. 1) with a cognitive purpose, i.e. to have students learn and remember better [15]. We acknowledge that the cognitive effects of vertically integrated curricula are important, but propose to view vertical integration from a broader perspective, that has a specific contextual component, a motivational component, and an identity component. Vertical integration may be still considered a curricular design matter, but we propose to extend its principles beyond the formal curriculum, offering significance for the continuum of education, professional development and practice.

With this article we aim to draw attention to this broader perspective.

### Vertical integration and context theory

Koens has studied the significance of context for learning and suggested that contextualized learning can be distinguished in three dimensions: a semantic or cognitive dimension (learning of science embedded in professional problems, such as in problem-based learning), a physical dimension (learning in a physical environment resembling the environment of future retrieval, as suggested by cognitive psychologists), and a commitment or affective dimension (learning by interacting with the context because of duties and responsibilities) [16]. While careful studies should still confirm this theory, it was suggested that the commitment dimension of context is more powerful for learning than the semantic or physical dimension [17, 18]. Contextualizing learning appears useful, but providing only a contextualized learning environment in the classroom, and even observation or shadowing in a clinical setting alone seems less powerful than engagement with duties in patient care. When vertical integration is predominantly regarded a reorganized curriculum structure, its implementation in practice might stay limited to curriculum alterations that address the cognitive and physical dimension. A curriculum that only provides cognitive links with clinical practice, or opportunities to observe clinical practice lacks the power of student engagement in patient care, i.e. of the increase of clinical responsibilities for students in the course of a curriculum [19].

### Vertical integration and motivation theory

The commitment (or affective) dimension for context for learning speaks to its motivational nature. Medical curricula have not often explicitly been designed for a motivational purpose [20]. Yet motivation in students may be the most important factor that determines a school’s success, and the question is therefore: can curriculum structure affect student motivation? Chen et al. found that students with a mastery goal orientation [21] perceived the clinical learning environment to be inviting and offering opportunities for active engagement and learning [22]. While these students already show motivation, the affordances in the environment must be present, arguably offered by the curriculum and therefore affording this motivation to grow. Those affordances may well be the opportunities that require a commitment to contribute to patient care. Lave and Wenger’s legitimate peripheral participation model supports the motivational effect of student engagement in a community of clinical practice, if even with small contributions [23]. A curriculum design that allows for such engagement may particularly enhance intrinsic motivation, which is stimulated by feelings of competence, autonomy and relatedness [24]. Early, small but significant tasks in health care, building up to tasks with greater responsibility later in the curriculum may serve that motivational function. Vertically integrated curricula may be particularly successful if not only early exposure to patients and the clinical environment is organized, but when even early learners are entrusted with small contributions to health care. That requires a social environment that affords these opportunities, and clinical teachers who discern these and stimulate learner agency to grasp the opportunities. If they do not view VI as a philosophy of education and do not feel the necessity to guide trainees in their participation in the professional community, including giving them increasing responsibility, the benefits of VI may stay limited. Entrustment decisions for small tasks are likely to boost an early learner’s motivation.

### Vertical integration and individual and social identity development

Vertical integration is likely to affect students’ professional identity formation. Identity formation is considered to happen simultaneously at individual and collective levels. Identity development at the individual level shows developmental stages. Moving from one stage to the next is thought to happen through ‘crises’, provoked by experiences and challenges faced [25]. In curricula with a gradual increase in clinical responsibilities trainees are longitudinally challenged to take steps in responsibilities in patient care. These are often the moments of crises, important for identity formation. Simultaneously, at the social level the trainees gradually move from peripheral to full participation in the community of practice. They learn to act within the community of doctors by internalizing values and norms. Van den Broek et al. studied professional identity formation in a vertically integrated curriculum that builds up to a final year deliberately designed to have students experience the responsibilities of a junior doctor (called ‘semi-physician’) under supervision. The authors found support for the notion that this curricular structure fosters students’ social identification with the community of doctors, and generates a genuine team-member sensation [26, 27].

### Vertical integration and the transition to practice

A vertically integrated curriculum, operationalized by Wijnen-Meijer et al. [28] as (i) provision of early clinical experience; (ii) integration of biomedical sciences and clinical cases; (iii) long clerkships during the final years of training; and (iv) fostering of increasing levels of clinical responsibility within undergraduate training, was found to support students’ capability to work independently, to solve medical problems, to manage unfamiliar situations, to prioritize tasks, to collaborate with others, to estimate when they need help, and to reflect on their activities, in comparison with medical students from a not vertically integrated curriculum. There was no measurable difference in knowledge and skills [29]. This may suggest the cognitive effects of vertical integration are limited, and the most important benefits for learners are in other domains. While replication of this study would be good, in a different study graduates from a vertically integrated curriculum appeared to make definitive career choices earlier, need less time and fewer applications to obtain residency positions and feel more prepared for work and postgraduate training [28].

### Vertical integration and the continuum of education and practice

Vertical integration not only offers early acquaintance with clinical experience, it also stresses the continued need to keep learning scientific backgrounds while acting in patient care. This extends beyond classroom learning in basic sciences in undergraduate education. Undergraduate, postgraduate and fellowships are those programs in medicine. Ten Cate & Carraccio have recently advocated to de-emphasize the strict transitions between these phases and unsupervised practice, and to view medical training as starting in medical school but really never ending until retirement [30]. Licensing and specialty certification are significant moments of transition in formal responsibility and privileging, but the acquisition of knowledge and skill and development of competence, and the formation of identity are more gradual and never fully finished. The continuum of training and practice, viewed from a distance, is always a mix of learning and practice, since the entrance in the clinical workplace with a white coat, until practice ceases. From this perspective, vertical integration is always present in a professional’s career. The continuum starts with classroom learning and little engagement in practice, while in clerkships, residency and fellowship, the learning component shifts to workplace learning. Learning is not finished after licensing or certification; there may even remain a need for supervision in the early years of ‘unsupervised’ practice. This was acknowledged in a recent study among veterinarians [31] but there is no principle difference with medicine.

## Conclusion

A regularly cited definition of vertical integration is “the integration between the clinical and basic science parts of the curriculum” [12]. This definition risks to limit the concept to a rearrangement of teaching topics that still may remain fragmented and reduced to curricular scheduling without the intended outcome of learning, i.e. improved patient care [13]. We propose to define vertical integration as “a deliberate educational approach that fosters a gradual increase of learner participation in the professional community through a stepwise increase of knowledge-based engagement in practice with graduated responsibilities in patient care”. Vertical integration in medical training is a philosophy of maturation and engagement with the profession, or, as Bloom and Cruess & Cruess have formulated for identity formation, *coming to think, feel and act as a physician* [32, 33], rather than merely a curricular arrangement. The authors of the current perspective article have used this philosophy in their work as medical educators [34]. Research on the effects show promising results [27], on the basis of which they now propose the new, more comprehensive definition. Vertical integration merges learning and practice, which does not stop at licensing or certification.

## Data Availability

Not applicable.

## References

[1] Harden RM, Sowden S, Dunn WR (1984). Educational strategies in curriculum development: the SPICES model. Med Educ.

[2] Miller G, Abrahamson S, Cohen IS, Graser HP, Harnack RS, Land A (1961). Teaching and learning in medical school.

[3] Brauer DG, Ferguson KJ (2015). The integrated curriculum in medical education: AMEE guide no. 96. Med Teach.

[4] Williams G (1980). Foreword, in Western Reserve’s experiment in medical education and its outcome.

[5] Barrows H, Tamblyn R (1980). Problem-based learning.

[6] Cox E (2015). Coaching and adult learning: theory and practice. New Dir Adult Cont Educ.

[7] Godden D, Baddeley A (1975). (1975) context-dependent memory in two natural environments: on land and underwater. Br. J. Psychol..

[8] Schmidt HG, Norman GR, Boshuizen HPA (1990). A cognitive perspective on medical expertise: theory and implications. Acad Med.

[9] Norman G (2009). Teaching basic science to optimize transfer. Med Teach..

[10] Kirschner P, Van Merriënboer JJG, Good TL (2008). Ten Steps to Complex Learning A New Approach to Instruction and Instructional Design. 21st century education: A reference handbook.

[11] Sweller J, Van Merriënboer JJG, Paas F (2019). Cognitive architecture and instructional design: 20 years later. Educ Psychol Rev.

[12] Dahle LO, Brynhildsen J, Behrbohm-Fallsberg M, Rundquist I, Hammar M (2002). Pros and cons of vertical integration between clinical medicine and basic science within a problem-based undergraduate medical curriculum: examples and experiences from Linköping. Sweden Med Teach.

[13] Benbassat J, Baumal R (2007). Viewpoint: a proposal for teaching basic clinical skills for mastery: the case against vertical integration. Acad Med.

[14] Wijnen-Meijer M, Ten Cate OTJ, Rademakers JJDJM, Van der Schaaf M, Borleffs JCC. The influence of a vertically integrated curriculum on the transition to postgraduate training. Med Teach. 2009:e528–32. 10.3109/01421590902842417 19909031

[15] Ten Cate O (2007). Medical education in the Netherlands. Med Teach.

[16] Koens F, Mann KV, Custers EJFM, ten Cate OTJ (2005). Analysing the concept of context in medical education. Med Educ.

[17] Koens F, Ten Cate OTJ, Custers EJFM (2003). Context-dependent memory in a meaningful environment for medical education: in the classroom and at the bedside. Adv Health Sci Educ.

[18] Koens F (2005). Vertical integration in medical education - studies on the required basic science knowledge and the concept of context. Doctoral dissertation.

[19] Ten Cate O, Snell L, Mann K, Vermunt J (2004). Orienting teaching toward the learning process. Acad Med.

[20] Kusurkar RA, Croiset G, Mann K, Custers E, Ten Cate O (2012). Have motivation theories guided the development and reform of medical education curricula? A review of the literature. Acad Med.

[21] Pintrich PR (2000). An achievement goal theory perspective on issues in motivation terminology, theory, and research. Contemp Educ Psychol.

[22] Chen HC, Ten Cate O, O’Sullivan P, Boscarding C, Eidson-Ton WS, Basaviah P, Woehrle T, Teherani A (2016). Students’ goal orientations, perceptions of early clinical experiences and learning outcomes. Med Educ.

[23] Lave J, Wenger E (1991). Situated learning: legimate peripheral participation.

[24] Ryan RM, Deci EL (2000). Self-determination theory and the facilitation of intrinsic motivation, social development, and well-being. Am Psychol.

[25] Jarvis-Selinger S, Pratt DD, Regehr G (2012). Competency is not enough: integrating identity formation into the medical education discourse. Acad Med.

[26] Van den Broek S, Querido S, Wijnen-Meijer M, Van Dijk M, Ten Cate O (2020). Social identification with the medical profession in the transition from student to practitioner. Teach Learn Med.

[27] Ellemers N, Haslam SA, Turner JC, Reynolds KJ, Van Lange PAM, Kruglanski AW, Higgins ET (2011). Social identity theory. Handbook of theories of social psychology.

[28] Wijnen-Meijer M, Ten Cate OTJ, Van der Schaaf M, Borleffs JCC (2010). Vertical integration in medical school: effect on the transition to postgraduate training. Med Educ.

[29] Wijnen-Meijer M, Ten Cate O, Van der Schaaf M, Harendza S (2013). Graduates from vertically integrated curricula. Clin Teach.

[30] Ten Cate O, Carraccio C (2019). Envisioning a true continuum of competency-based medical education, training and practice. Acad Med.

[31] Duijn C, Bok H, Ten Cate O, Kremer W (2019). Qualified but not yet fully competent: bridging the gap between education and clinical practice. Vet Rec.

[32] Bloom S (1963). The process of becoming a physician. Ann Am Acad Political Soc Sci.

[33] Cruess RL, Cruess SR, Boudreau JD, Snell L, Steinert Y (2014). Reframing medical education to support professional identity formation. Acad Med.

[34] Ten Cate O, Borleffs J, Van Dijk M, Westerveld T (2018). Training medical students for the twenty-first century: rationale and development of the Utrecht curriculum “CRU+”. Med Teach..

